# Editorial: Immune dysregulation in inborn errors of immunity

**DOI:** 10.3389/fped.2023.1329023

**Published:** 2023-11-30

**Authors:** Eveline Y. Wu, Amir H. Shahlaee, Mildred Kwan

**Affiliations:** ^1^ Division of Rheumatology, Department of Pediatrics, University of North Carolina, Chapel Hill, NC, United States; ^2^ Division of Allergy/Immunology, Department of Pediatrics, University of North Carolina, Chapel Hill, NC, United States; ^3^ Dr. Helbing Allergy and Asthma Associates LTD, Annandale, VA, United States; ^4^ Division of Rheumatology, Allergy, and Immunology, Department of Medicine, University of North Carolina, Chapel Hill, NC, United States

**Keywords:** inborn errors of immunity, immune dysregulation, autoimmunity, immunodeficiency, primary immunodeficiencies

**Editorial on the Research Topic** Immune dysregulation in inborn errors of immunity

The number of inborn errors of immunity (IEI) is growing rapidly (1). They can be challenging to diagnose given the expanding and variable clinical phenotypes (2, 3). Specifically, there is an increasing recognition that immune dysregulation can be an initial or predominant manifestation of a substantive portion of IEIs (4, 5). Immune dysregulation can also have a large impact on disease treatment, monitoring, and outcomes. Significant progress has been made in the elucidation of molecular and cellular mechanisms underlying the immune dysregulation of IEIs. Tremendous improvement has also been made in the management of IEIs with availability of therapies expanding to include biologics and small molecular inhibitors. Despite progress, more understanding is needed to tailor the use of immunomodulatory therapy in IEIs.

This Research Topic “Immune Dysregulation in Inborn Errors of Immunity” highlights recent advances in the understanding of the prevalence, mechanisms, spectrum of manifestations, and management of immune dysregulation in IEIs. Gagne et al. provide a concise review of how Mendelian type I interferonopathies can masquerade as non-Mendelian autoimmune disorders like systemic lupus erythematosus (SLE) and dermatomyositis, which are also associated with increased type I interferon (IFN) expression. Similarly, Hetrick and Oliver described how autoinflammatory bone disorders characterized by sterile osteomyelitis can be seen with both monogenic forms including Majeed syndrome and deficiency of the interleukin-1 antagonist (DIRA) and the more common sporadic form chronic nonbacterial osteomyelitis (CNO) or chronic recurrent multifocal osteomyelitis (CRMO). Other autoimmune manifestations of IEIs include, but are not limited to, autoimmune cytopenia, endocrinopathies, inflammatory bowel disease, neutrophilic dermatoses, arthritis, and vasculitis as illustrated by the case of activated phosphoinositide 3-kinase delta (PI3Kδ) syndrome (APDS) from Sood et al.

The highly variable clinical manifestations of IEIs can contribute to diagnostic delays, which in turn, can lead to poorer outcomes. Autoimmunity and autoinflammation can negatively impact morbidity and mortality and are an independent prognostic factor for death among individuals with IEIs (5). Immune dysregulation may also contribute to the increased risk of malignancy observed in IEIs as with the APDS case presented by Sood et al.

Improving awareness and early recognition of IEIs is also critical because making a diagnosis can impact treatment and surveillance. There may be disease-specific treatments available like leniolisib, an oral selective PI3Kδ inhibitor, for APDS as discussed by Sood et al. Because type 1 IFN work through the janus kinase/signal transducer and activator of transcription (JAK/STAT) pathway, Gagne et al. also consider the increasing use of JAK inhibition in autoinflammatory IFN-mediated monogenic diseases. The study by Berrueco et al. showed mycophenolate mofetil may be an effective and safe treatment for pediatric patients with autoimmune cytopenia and IEI, even cases of autoimmune cytopenia refractory to first-line therapies like corticosteroids and intravenous immunoglobulin (IVIG).

These articles also nicely illustrate the need for a multidisciplinary approach to the immune dysregulatory manifestations in IEIs as there are contributions from Rheumatology (inteferonopathies and pediatric autoinflammatory bone disorders), Hematology (autoimmune cytopenias), and Allergy/Immunology (APDS). The conditions covered in this topic represent only a fraction of disordered immune conditions in IEIs and should be considered in more detail in the future.

The recognition of immune dysregulation in IEIs continues to increase as the number of patients impacted expands. There is a clear need for ongoing investigation into the epidemiology and pathophysiology for clinical manifestations and the risk factors for developing immune dysregulation as well as a collaborative multidisciplinary approach to improve diagnosis and management of this population. Our hope is that this topic has piqued the interest of researchers and clinicians in this area to provide better understanding, care, and outcomes to these patients.

Lastly, we would like to thank the reviewers for their time and valuable assessments. This important collection of articles would not be possible without their contributions.
